# Real-Time Safety Evaluation for Slope during Construction Using Numerical Forecast and Sensor Monitoring Platform

**DOI:** 10.3390/s18092978

**Published:** 2018-09-06

**Authors:** Sherong Zhang, Dejun Hou, Chao Wang, Xuexing Cao, Fenghua Zhang, Fei Pan, Chengbo Du

**Affiliations:** 1 State Key Laboratory of Hydraulic Engineering Simulation and Safety, Tianjin University, Tianjin 300072, China; tjudam@126.com (S.Z.); hdj@tju.edu.cn (D.H.); zfh101207@163.com (F.Z.); 2 School of Civil Engineering, Tianjin University, Tianjin 300072, China; 3 State Key Laboratory of Water Resources and Hydropower Engineering Science, Wuhan University, Wuhan 430072, China; 4 Huaneng Lancang River Hydropower INC., Kunming 650051, China; 5 Shanghai Municipal Engineering Design Institute (Group) Co., Ltd. Shanghai 200092, China; pfei19@126.com; 6 Yalong River Hydropower Development Company, Ltd., Chengdu 610051, China; duchengbo@ylhdc.com.cn

**Keywords:** slope engineering, real-time safety evaluation, real-time system, construction schedule, information technologies

## Abstract

Geology uncertainties and real-time construction modification induce an increase of construction risk for large-scale slope in hydraulic engineering. However, the real-time evaluation of slope safety during construction is still an unsettled issue for mapping large-scale slope hazards. In this study, the real-time safety evaluation method is proposed coupling a construction progress with numerical analysis of slope safety. New revealed geological information, excavation progress adjustment, and the support structures modification are updating into the slope safety information model-by-model restructuring. A dynamic connection mapping method between the slope restructuring model and the computable numerical model is illustrated. The numerical model can be generated rapidly and automatically in database. A real-time slope safety evaluation system is developed and its establishing method, prominent features, and application results are briefly introduced in this paper. In our system, the interpretation of potential slope risk is conducted coupling dynamic numerical forecast and monitoring data feedback. The real case study results in a comprehensive real-time safety evaluation application for large slope that illustrates the change of environmental factor and construction state over time.

## 1. Introduction

**Figure 1:**
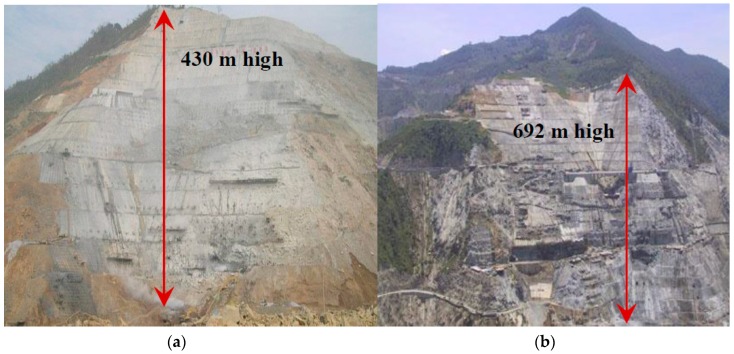
High steep slopes in China related to reservoir safety (**a**) Longtan high steep slope, China (**b**) Xiaowan high steep slope, China.

The high steep slopes are confronted with challenging problems and are closely related to reservoir safety, e.g., 430 m high Longtan slope (Guangxi province, China) and 692 m high Xiaowan slope (Yunnan province, China), as shown in Figure 1, with the main characteristics, including large scale, long engineering life, high safety standard, and the difficulties that are encountered in design, construction, and operation [1]. Clearly grasping the geological state of the whole slope is almost impossible before construction. Currently, there are mainly three methods of mapping large-scale slope safety and hazards: qualitative methodologies, statistical methodologies, and geotechnical model-based methodologies [2]. But, research work pays more attention to the static evaluation method at a certain moment, for example, general limit equilibrium method and finite element method [3,4,5], probabilistic stability analyses [6,7], and deterministic analyses [8,9]. Generally, previous simulations have commonly failed to consider the time-varying changes in geological and construction conditions. Real-time safety analysis and evaluation during the whole process of slope construction are still limited.

In practice, time-dependent construction safety has become critical technical problem due to the time-varying geotechnical environments and the complex construction process [10,11]. Firstly, the geological updating situation is restricted by the geological conditions and the depth of the geological prospecting work in different design stages. It is distorted to still adopt former values without considering new revealed information to real-timely evaluate the slope engineering safety during construction. Secondly, part of the slope safety information is prevented from being updated in real time, few data on visualization tools in construction exist in real-time [12]. Thirdly, once the construction plan needs to be modified, the original structure analysis models also need to undergo the corresponding changes. Therefore, a new model needs to be established and updated in line with the new situation. These limitations of current practices can become a bottleneck for fast and accurate real time safety evaluation on a busy hydraulic construction site.

There is a pressing need for a higher degree of assistance from new automated technologies, tools, and approaches, while taking into account changes during the slope construction process [13]. Nowadays, field monitoring is an important means to evaluate the safety state of slopes, and numerous studies have been conducted on the interpretation of slope safety based on monitoring information. Structural health monitoring (SHM), which is a comprehensive instrumentation of structures and environments [14,15], is widely recognized as a crucial element of slope construction safety evaluation. Common instruments and measuring indexes in slope monitoring are summarized according to [16]. A systematic method of slope safety evaluation is presented utilizing multi-source monitoring information with Bayesian networks [17]. GIS (Geographic Information System) -based method and coupling methods [18,19] have been attempted slope safety evaluation and warning. ANN (Artificial neural network) and MR (multiple regression) models are also employed [20]. A multi-method approach combination of geotechnical field and laboratory works, GIS, and mechanical stability analysis for the stability assessment of slopes is presented [21]. Visualization technology and the safety information model are also used in construction safety evaluation [5,22,23]. BIM (Building Information Modeling) technology has been used in construction safety management research for the whole life cycle [24]. BIM-based model is proposed as an integrated tool to present real-time visualization safety status of related components under changing conditions [11]. Early warning systems, including the setting of appropriate alarm levels for the rockslide, are described by using expert judgment and forecasting methods [25]. In addition, gathering and processing construction resource data in real-time and visualizing relevant safety and activity performance information are presented in literature [12].

But, the present new technologies for slope safety analysis also have some limitations: (1) considering its special geo-environment and engineering position, some instruments must be effective after slope excavation and remedy, so real-time automatic monitoring is a big challenge; (2) historical monitoring data has generally been limited to identify and mitigate only one aspect of construction safety [26], rather than holistic assessment; and, (3) it is mainly a feedback approach and ignore numerical predictions, which thus prevents a dynamic construction schedule adjustment from being available.

In this regard, this paper extends previous studies and the primary differences between the present work and the previous study include: (1) a model and methodology for real-time slope safety evaluation during the construction for large-scale slope is proposed; (2) the slope safety assessment is achieved using both monitoring information and updated numerical results; and, (3) time-dependent evaluation system coupling analysis, forecast, and feedback is developed for large scale slope construction state.

## 2. Model and Methodology

**Figure 2:**
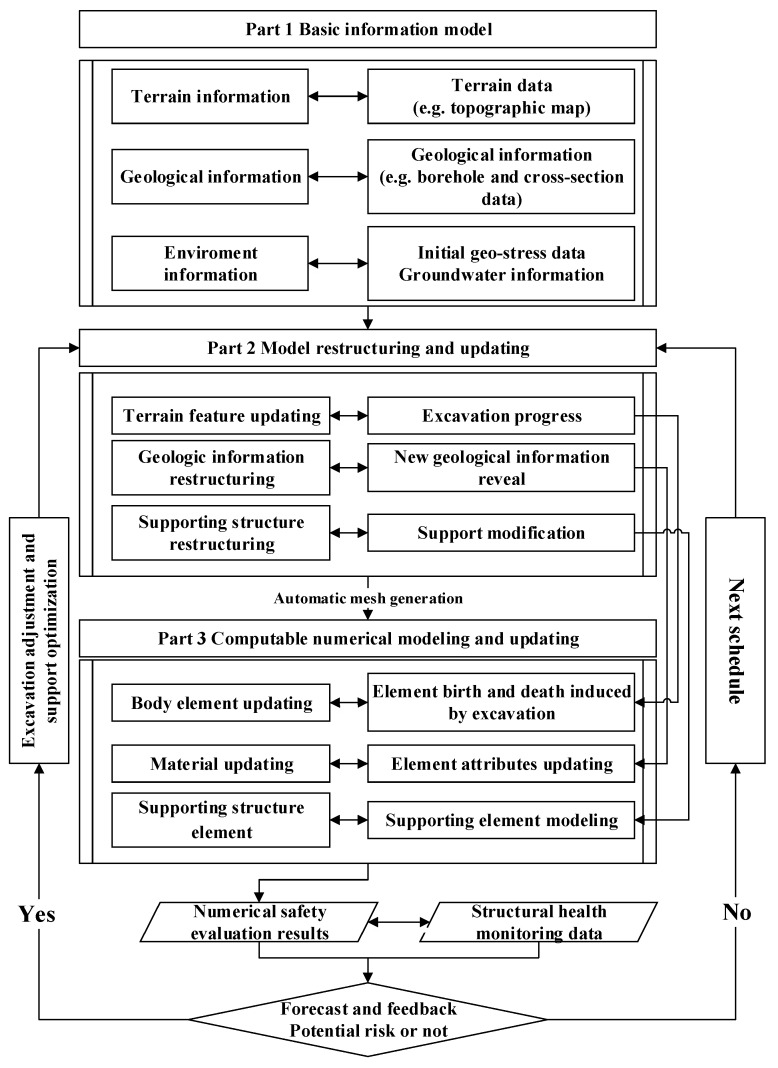
Diagram of the architecture of proposed safety information model consisting of key elements.

The real-time numerical results and sensing data stored in field monitoring database are both employed to conduct on the interpretation of slope safety. Diagram of the architecture of proposed safety information model is provided in Figure 2 to show the architecture of the proposed model and method.

It mainly has three key parts: (1) basis information modeling; (2) model restructuring and updating; and, (3) numerical modeling and updating. It should be noted that the sensing/monitoring data are required to retrieve from monitoring and inspection center database. This data repository is developed for the polytropic data collection.

Firstly, a primary three-dimensional (3D) model is established according to the geological survey. Automatic monitoring units and instruments have already been installed during the geological survey and the construction progress. They have been operated in a stable wireless manner to collect the basic information and have managed several monitoring items [27], as shown in Table 1. All of the information is collected into the data repository.

Secondly, there must be many excavation progress adjustments or the support structures modification during the constructions, which induce the 3D model restructuring. To make the real-time evaluation of slope safety during construction, the adjustments or modification must be mapped into the numerical model according to their location through the conversion relations between the model’s coordinate system and geodetic coordinate system. Otherwise, the next construction schedule would be carried on.

Thirdly, numerical modeling and updating can be made through automatic mesh generation method illustrated in Section 2.3 in detail. It should be noted that the slope safety evaluation is made by coupling dynamic numerical forecast and monitoring data feedback, which means that the dynamic numerical forecast results from the numerical method are employed to reflect the regional safety. If the numerical evaluation results are inconsistent with monitoring results from observational points, the basic parameters should be recalculated and recovered from the monitoring curves to solve the identification models. Finally, the numerical forecast results and the monitoring information are consistent with each other at the observational points.

**Figure 3:**
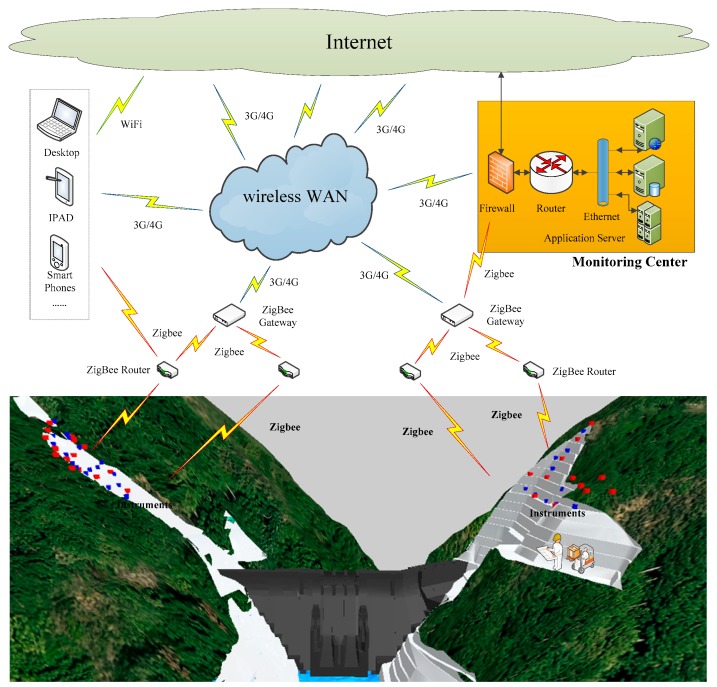
Schematic view of the structural health monitoring system. (WAN means Wide Area Network).

Because the instrumentation and monitoring technology and system of slope stability are relatively mature [27,28], our attention is concentrated on the real-time numerical model and system implementation. Figure 3 gives the schematic view of the structural health monitoring system that is used in this paper.

### 2.1. Basic Information Modeling

A basic information model for slope engineering contains initial geostress and groundwater information, terrain information, geologic information, et al. It is easily obtainable that collecting spatial information on environmental factors with a focus on digital elevation model (DEM) [29], geology and soils, geomorphology, land use, and elements at risk [30]. A 3D terrain model is established based on digital elevation model. Triangular irregular network (TIN) [31] is used to obtain the initial geological interfaces, including ground surface, interface of rock stratum, fault surface, and artificial excavation surface. 3D geo-model technology relies heavily on large numbers of borehole and cross-sectional data obtained from geological investigation stage. The data interpolation method, such as Kriging interpolation [32] should be used and a curved surface fitting method should be applied to establish geological model. The “aperture-deforming” method using the hollow inclusion cell instrument is adopted for the in-situ stress measurement [33,34] to obtain the three-dimension geo-stress at the measuring point that is based on the relationship between the pitch deformation and the geo-stress.

Then, a three dimensional deformation inversion analysis [35] can be conducted to determine the ground stress field at various depths. All types of information related to the slope safety analysis during the construction period should be stored in the database as a 3D data structure, which means the geometric model data required for safety information visualization and relevant safety information can be quickly retrieved in the following system platform.

### 2.2. Model Restructuring and Updating

**Figure 4:**
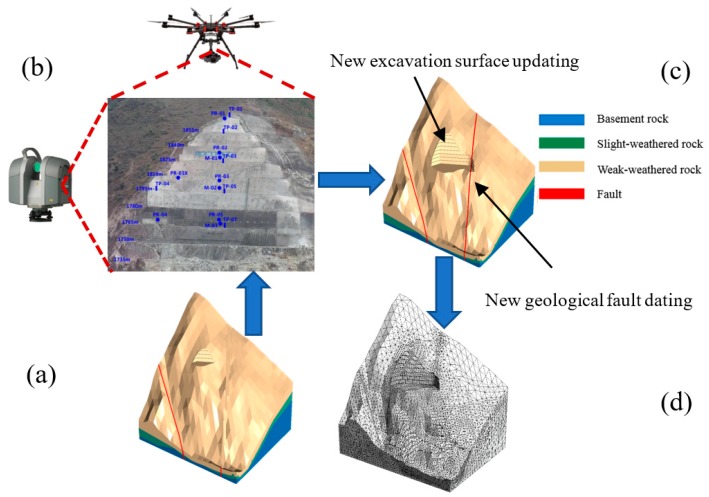
Diagram of model restructuring and updating of the slope construction. (**a**) 3-dimensional slope structural model in previous state (**b**) Real-time construction state obtained through drone and total station (**c**) 3-dimensional model restructuring and updating of the real-time construction state (**d**) Numerical calculation model updating.

The real-time construction state and the progress of the excavation will be inevitably impacted with the emergence of abrupt geological changes or bad weather, e.g., rainfall, so that the restructuring and updating of slope information model should be valued. Model restructuring and updating technology are presented and established for a dynamic connection between the slope structural system and the construction process information so that three-dimensional slope structural models corresponding to real-time construction state can be generated rapidly and automatically. Figure 4 shows the process of model restructuring and updating of the slope construction, and the details are illustrated, as below.

#### 2.2.1. Terrain Features Restructuring

A top-down excavation method is adopted in slope engineering, and the excavation body on each floor is divided into several separated item projects. Assistance from new automated technologies, tools, and approaches, e.g., unmanned aerial photography technology and electronic total station, are employed for investigating excavation body. Spatial information of the excavation body location (x, y, z) or supporting structure, time information of the construction step, are restructured in the 3D terrain model so that the terrain model are updated.

#### 2.2.2. Geologic Information Restructuring

Geologic information restructuring mainly rely on real monitoring information and geology that are exposed during the construction rather than previous estimation. The excavation condition keeps changing during slope construction so that the original slope geologic model is no longer applicable. Both conditions significantly impact the engineering real-time safety. With appropriate instruments, changes in slope properties, such as soil stresses, pore water pressures, and crack development can be measured [16], and got from monitoring items database.

Many new methods have been emerging and tentatively used in geotechnical engineering to analyze and evaluate the monitoring data so that more and more geologic information can be got with the development of intelligence science and mathematics theory. Then, the new geological defects, e.g., joint fissures and faults and environmental induced geologic information can be collected into the database and finally mapped into the numerical model with the mapping method in [5], which will not be repeated here.

#### 2.2.3. Supporting Structure Model Restructuring

Supporting scheme modification is usually taken during the slope construction. The support progress information comes from the real support schedule rather than the design schedule. But, the construction schedule update does not take all temporary structural supports into consideration, security administrators mainly focus on the structural safety state under the condition of the permanent support and the system adopted the mechanical equivalent principle to consider other pre-support and temporary support to reduce the complexity and improve the efficiency of real-time computing.

Supporting restructuring and modification information can be mapped into the numerical model with the mapping method in [5], which will not be repeated here.

### 2.3. Numerical Model Updating

The method to reconstruct and update your model with new monitoring information is the core step in our platform. In the method, real-time restructured information is mapped into the dynamic numerical simulation model and updating information into the database, rather than visual management in the conventional 3D model [5]. Mapping method describes how the excavation progress and geometrical information of each separated item project should be mapped to the numerical model. The implementation method is previously illustrated in literature [5], which will not be repeated here. Generally, there is a dynamic connection between the 3D slope restructuring model and the numerical model, so that the computable numerical model can be generated rapidly and automatically. This progress mapping method is also adopted to support structures, and the material properties modification of related elements is realized with field variables. For example, the method of geology information updating is summarized below: Firstly, nodes within the location of the new geological area of numerical model are searched out by crafting a program, then a new geological set is created to group all of the corresponding model elements. Thirdly, new material parameters are set to geological conditions. The numerical model updating process is presented by secondary development to the numerical analysis program to simulate the addition and modification of supporting structures.

## 3. System Implementation

**Figure 5:**
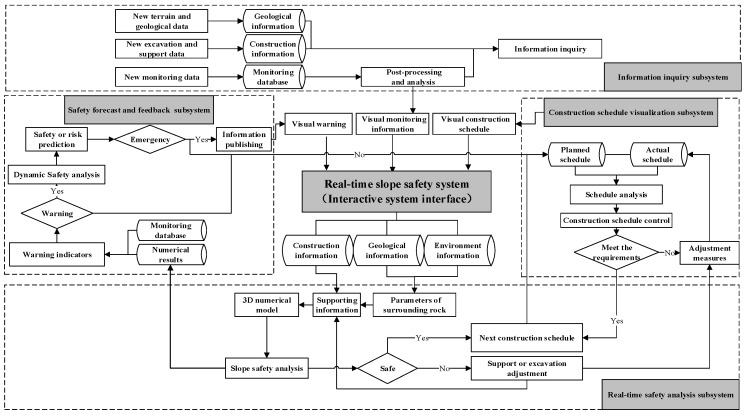
System architecture for time-dependent slope construction.

The object-oriented programming method is used to tailor an integrated working environment for slope engineering construction planners. Figure 5 displays the framework of the real-time safety evaluation system including four subsystems, construction information inquiry subsystem, construction schedule visualization subsystem, real-time safety analysis subsystem, and safety forecast and feedback subsystem based on numerical results and monitoring data. Figure 5 covers the workflow throughout the whole construction project. A variety of tools are furnished by the platform. To ensure a smooth transition between different subsystems and applications, each module in the system shares the same database and this database-based approach is very convenient for data renewing. Moreover, our system is designed to be a stand-alone application platform, which takes advantages of popular commercially available platforms. ABAQUS 13.0 has been chosen as the safety analysis subsystem for some modules to get the real-time safety information during the slope construction so that the numerical simulation methods are traditionally based on ABAQUS 13.0. Inversion analysis method [36] is employed to simulate the geo-stress of the slope in numerical analysis. Meanwhile, to make it easier for engineers to analyze the amounts of monitoring data, the system contains several intelligent analysis methods, including time series analysis, grey mathematics, neural network, and so on.

The system does not directly collect safety monitoring data, while this work is made by an automatic monitoring system from monitoring and inspection center in each hydraulic construction site and monitoring data can also be retrieved automatically from the connection of shared database.

## 4. Case Study

**Figure 6:**
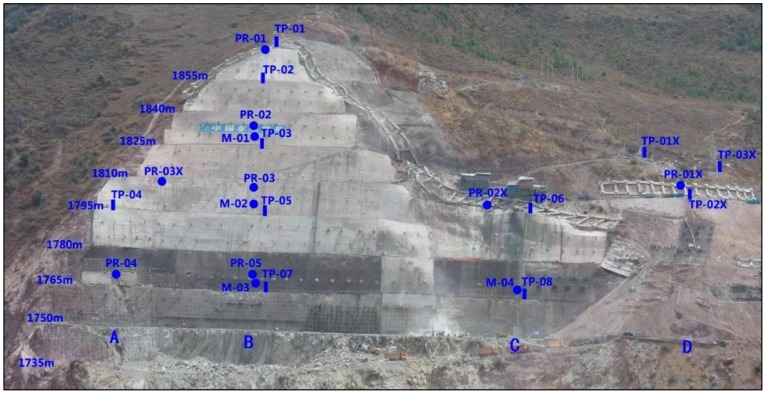
Monitoring allocation in typical slopes of Huangdeng.

Huangdeng dam in Yunnan province, China, is the tallest compacted concrete gravity dam under construction in the world. Its high steep slopes are closely related to dam and reservoir safety. The maximum height of the right bank slopes in the Huangdeng hydropower station is approximately 178 m, the horizontal range is approximately 265 m, and the natural slope grade is 40°–55°, with a blocky structure toward the downstream. The rock masses that are caused by unloading relaxation are more widely distributed, with 70–150 m width and 20–70 m thickness. The volume of the rock mass is approximately 1.2 million to 1.5 million m^3^. Figure 6 displays monitoring allocation in typical slopes. Up to 20 April 2013, several monitoring instruments have been running above 1735 m, including 11 surface displacement monitoring instruments (TP-01~TP-08; TP-01X~TP-03X), four multiple position extensometers (M-01~M-04), and eight anchor dynamometers (PR-01~PR05; PR-01X~PR03X) in A–D areas. Taking the right bank slopes of Huangdeng hydropower station as an example, a real-time evaluation system of the slope engineering safety during the construction period is illustrated and recommendations on the follow-up construction schedule are provided.

**Figure 7:**
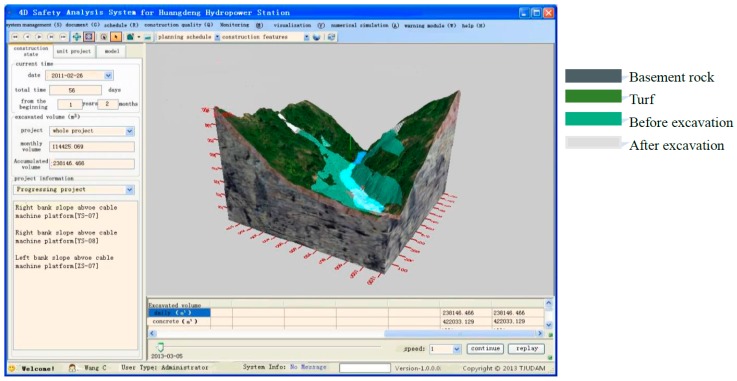
System interface for time-dependent slope construction (translated from Chinese Interface).

The slope safety information dynamic visual management system of a hydropower station during the construction period is developed in the Visual C#.NET programming environment using the framework Tao of OpenGL 3D graphics API (Application Programming Interface). Figure 7 shows the system interface, which contains seven steep slopes that are related to reservoir safety. During the construction of the slope engineering, the system was used to evaluate the real-time safety of the slope and it had been proved to be effective. Wavelet denoising methods [37] are used to eliminate systematic error and random error in monitoring data. Both the dynamic numerical safety evaluation results and monitoring data has been retrieved and read automatically from the database to analyze the real-time safety in Huangdeng slope.

### 4.1. Information Inquiry

**Figure 8:**
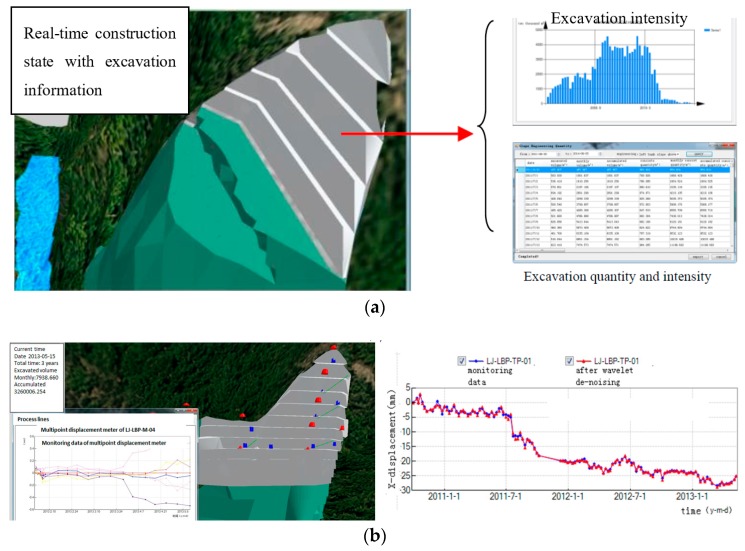
Interactive queries of information for real-time state of the slope. (**a**) Excavation quantity data; (**b**) Monitoring instrument and monitoring data curves.

In this subsystem, geometric information, geologic information, environment information, construction schedule information, monitoring information, as well as the safety information of the slope during the whole construction can be obtained. Geometric information, geologic information, and environment information come from the restructuring and updating 3D model in the database, and data interchange can be made from the platform. The construction progress at any time point during the construction period, including excavation quantity and excavation intensity, can be achieved. Visualization results of the excavation quantity of the construction information can get for the real-time state of the right bank slopes. Figure 8a shows the excavation quantity data for real-time state of the slope on 4 May 2013. It can be developed to capture the current state of the excavation progress, as well as the excavation intensity and excavation quantity that the project would need to attain over the next day or week. Interactive queries of monitoring information can be performed in terms of curves by clicking a monitoring point in the monitoring instrument of the 3D slope model, as shown in Figure 8b. Safety information of the slope mainly comes from the numerical simulation results and the monitoring data. Detailed illustrations are presented in the safety analysis subsystem.

### 4.2. Construction Schedule Visualization

The traditional method to express the construction schedule is a Gantt chart, which does not consider the construction status or composition of the site, and it can be very difficult for most workers or engineers to interpret due to the large number of activities that are present and the complexity of the relationships and the notations.

**Figure 9:**
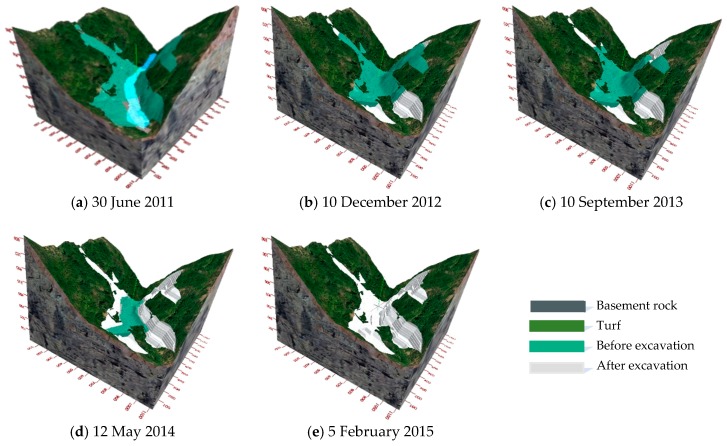
Visualization of the construction schedules for the slopes at different time (**a**) Construction features on 30 June 2011; (**b**) Construction features on 10 December 2012; (**c**) Construction features on 10 September 2013; (**d**) Construction features on 12 May 2014; and, (**e**) Construction features on 2 February 2015.

Our subsystem will generate snapshots of project progress for any given day based on the as-planned and as-built schedules. Alternative schedules can be created with the help of snapshots that are generated through the 3D model, such as the one shown in Figure 9, which affix visual information to daily or weekly milestones. In this way, differences between the planned and the real schedules can be visualized, and such snapshots can then be used as reports, thus making it easier for project participants to interpret schedule information and progress targets.

**Figure 10:**
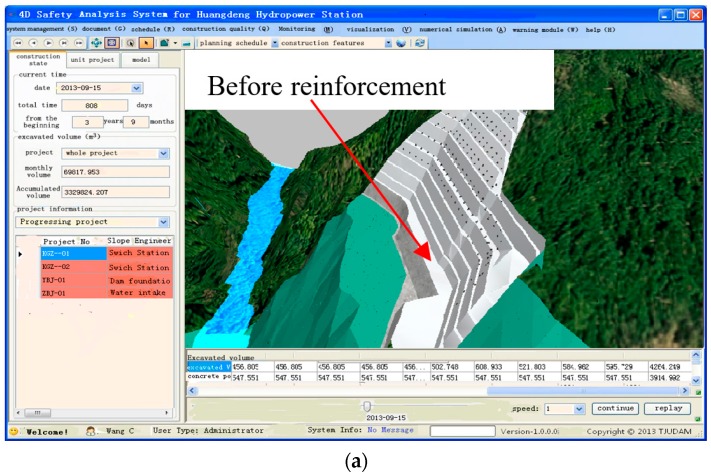

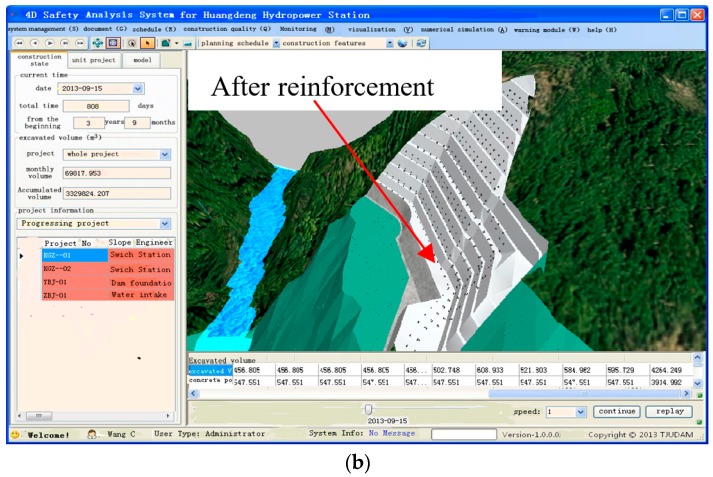
Visualization of the supporting schedules for the slopes. (**a**) Visualization of the supporting schedules before 10 September 2013; and, (**b**) Visualization of the supporting schedules after 10 September 2013.

The snapshots can be used to generate visual look-ahead targets. Similarly, with the help of snapshots generated, supporting structures, such as the one shown in Figure 10, can help to determine the support parameters of bolt as well as the supporting schedules from the database behind the visualization model. The subsystem above can be used to capture the current state of the supporting structures as well as the future visual state that the project would need to attain over the next week or month. This user-friendly operation mode enables planning of re-scheduling operations to be processed in a 3D environment and it facilitates the evaluation of alterative construction plans.

### 4.3. Real-Time Safety Analysis

The real-time safety analysis subsystem coupling construction schedule is the core content of the proposed system. Real-time safety analysis mainly relies on two aspects: numerical simulation results and potential trends from monitoring data. As mentioned, the change of the dynamic numerical model is accomplished by mapping the real-time state information of the slope into the database so that 3D structural analysis model of slopes can be generated rapidly and automatically with the mesh generation method that is based on Hypermesh 11.0. Then, numerical simulations are conducted on ABAQUS 13.0 coupled in the system and the point safety factors can be got.

**Figure 11:**
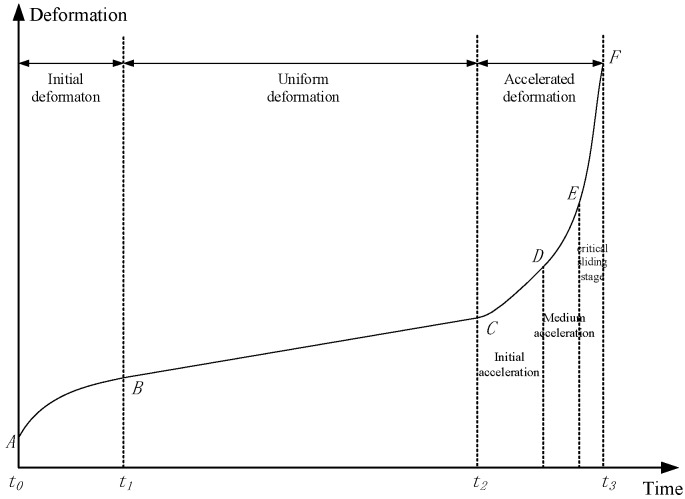
Different characteristics for evolution of slope deformation based on monitoring data analysis.

The criteria of safety assessment are normally from the design standards of the slope. The index of stability is a well-known safety factor. But, for the complexity of the slope engineering, slope stability evaluation cannot rely on a single method. The rigid limit equilibrium method is not employed in this research. A comprehensive evaluation models and method [38] are employed to make the safety assessment. A set of evaluation criteria was established for each adopted index, mainly refers to deformation, deformation rate, and safety factor in this subsystem. The acceleration before and after the landslide that is under critical sliding stage shows completely different characteristics, as shown in Figure 11.

**Figure 12:**
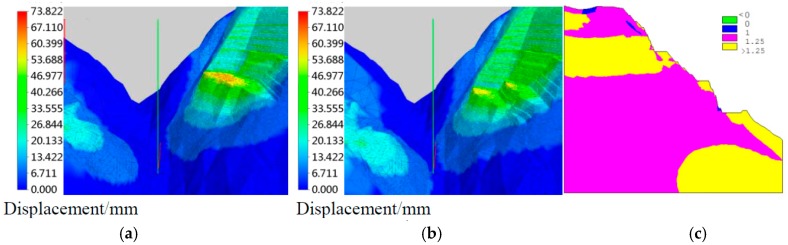
Numerical simulation results for real-time safety evaluation (**a**) Deformation of the slope at 4 May 2013; (**b**) Deformation of the slope at 10 September 2013; and, (**c**) Safety factor of slope section.

The predictable numerical results of displacement nephogram during the construction schedule are shown in Figure 12a,b. The safety factor is reached so that the safety factors of specific slope section are presented in Figure 12c. Furthermore, the numerical results and point safety factors can be validated at the observational points though monitoring results, which has proved to be effective in the actual projects.

### 4.4. Safety Forecast and Feedback

**Figure 13:**
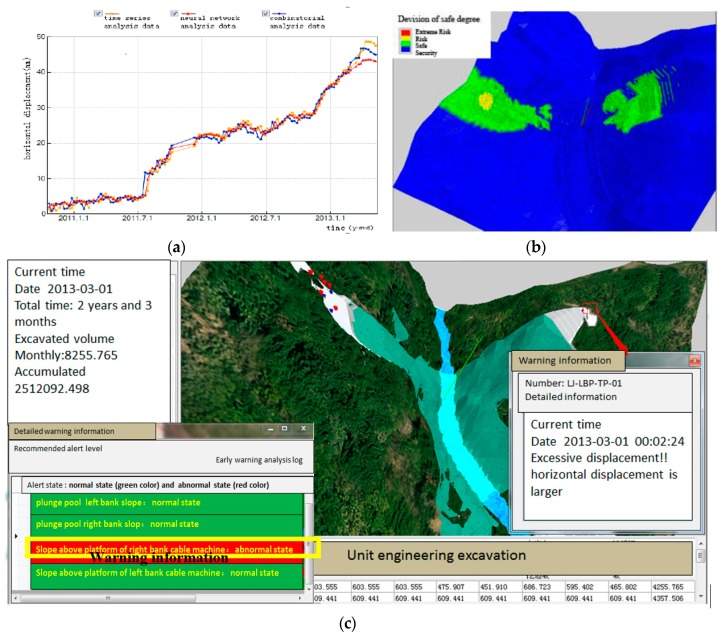
Early warning and feedback based on numerical results and monitoring data (**a**) Forecasting analysis of monitoring information with different methods; (**b**) Real-time safety analysis results determined from the numerical simulation; and, (**c**) Specific early warning information of slope area.

Analyzing the potential trends of monitoring data is another important way to indicate the real-time safety state of the slope [39], which can be achieved via monitoring data analysis. This can also be used as the important basis of slope safety assessment. Using the early warning and forecasting subsystem, the changes in the monitoring data and its trends can be determined based on a quantitative criterion. Changes in the displacement or seepage information inside of the slope are continuously monitored and the trends are predicted with the analysis of neural network. It will play a significant role in analyzing the development trend, predicting possible values, and conducting security monitoring to set up a corresponding monitoring model based on the monitoring data of the slope deformation, stress, seepage, etc. Different evolutionary patterns of displacements can be recognized through the analysis of the monitoring data to assess alert thresholds according to quantitative criterion in literature [40], as shown in Figure 13a.

Real-time safety factors and the devision of safe degree will be calculated based on the results from the numerical simulation, as shown in Figure 13b. If abnormal points appear in the construction safety information model, this system will trigger a warning for decision makers and construction participants, as shown in Figure 13c. Based on the thresholds of the monitoring data and the safety factor of the numerical results, the future state that the project will achieve over the next few days can be captured. Project participants can grasp the evolution law of the slope safety situation, along with the excavation process timely and accurately. A continuously and dynamically time-dependent analysis of the construction state for the slope is achieved.

Additionally, abnormal results are investigated in detail by clicking on the abnormal points in the system. Email and text message are employed to send the specific abnormal information rather than weekly or monthly paper reports, so that the updates can perform automatically.

## 5. Conclusions

A real-time safety evaluation method is less involved and still limited for mapping large-scale slope hazards. The slope safety information model is introduced into the time-varying safety analysis through the dynamic mapping method. A dynamic connection mapping method between the slope restructuring model and the computable numerical model is illustrated and the numerical model can be generated rapidly and automatically in database. The actual construction schedule of item projects can be tracked and mapped into numerical model dynamically. A database-based system of slope safety is developed to realize the real-time simulation and forecast the real-time construction state. This system has already experienced the test of Huangdeng slope construction process and it has been proven to be effective.

Using real-time slope safety evaluation method makes mapping large-scale slope hazards during the dynamic construction progress easier, but there is still a lot of work to do in the future. On one hand, this system is large enough to require expertise from many disciplines and it would take more resource to apply the methodology for multiple cases studies. On the other hand, the feedback analysis method from the monitoring data for real-time safety simulation analysis is still limited.

## Figures and Tables

**Table 1:** Slope health monitoring data in the database [27].

Number	Catalogue	Monitoring Item Name	Monitoring Method
1	Environment factors	Water overflow level meter	Electric
2		Underground water flow meter	Electric
3		Weather and river water level	Electric
4		Thermometer	Automatic
5		Fracture activity indicator	Electric
6	properties	Superficial surveying	Optical
7		Multipoint extensometer	Auto/manual
8		accelerometers sensors	Auto/manual
9		Reinforced steelbar strain gauge	Auto/manual
10		Borehole inclinometer	Electric
11		Joint meter	Auto/manual
12		Pore pressure transducer	Automatic
13		Soil pressure cell	Automatic
14		vibration sensors	Automatic
